# No Value of Routine Brain Computed Tomography 6 Weeks after Evacuation of Chronic Subdural Hematoma

**DOI:** 10.1055/s-0038-1673341

**Published:** 2018-12-10

**Authors:** Mehmet Turgut

**Affiliations:** 1 Department of Neurosurgery, Faculty of Medicine, Adnan Menderes University, Aydın, Turkey

Response to Letter to the Editor

I read with a great interest the article by Pedersen et al entitled “ *No value of routine brain computed tomography 6 weeks after evacuation of chronic subdural hematoma* .” 1 As stated by the authors, chronic subdural hematoma (CSDH) is a common neurosurgical condition, with an increased incidence and prevalence in elderly population. 2 Based on their findings, they concluded that routine postoperative control brain computed tomography scan performed 4 to 6 weeks after the evacuation of CSDH as a traditional approach has no clinical value. 1 Unfortunately, however, there are some important methodological and reporting problems that limit the scientific value of this article as follows:

1. Even today, some aspects of CSDH are not well studied and no consensus exists in the management of the patients with CSDH, although CSDH is a common neurosurgical condition. Surgical drainage is the gold standard treatment for the management of symptomatic CSDH, but there is no consensus yet regarding the optimal surgical technique, as mentioned by the authors. 1
2. The authors prefer using Glasgow coma scale (GCS) for the evaluation of their patients upon admission to the hospital and at discharge, but most authors in the literature have used the clinical grades described by Markwalder for classifying patients with CSDH. 3 4 5 I also think that GCS is not useful for this entity because the relevant score assigned for almost 83% of the hematomas in their series is between 13 and 15 on the GCS. 1
3. It is likely that there are 4 patients with bilateral CSDH among 198 patients in their series. Nevertheless, there is a serious confusion regarding the total number of patients in different parts of the article text: “ *202 patients* ” in Abstract section, while “ *202 hematomas* ” in Materials and Methods, and Results sections. 1 In particular, there is a similar disagreement in different columns for the total number of patients: 198 in some columns and 202 in the remaining columns, pending a clarification by the authors of the article.
4. Interestingly, there is a paragraph describing the details of statistical analysis in Materials and Methods section, including Mann–Whitney's *U* test and chi-square test, but there is no result with statistical analysis described in this section. Thus, the authors should clarify how they gave the redundant information in Materials and Methods section.
5. Moreover, there are several number adjectives which were misused within the article text such as “ *the majority of the CSDHs* ” used for 92 patients in Results section, “ *in several cases* ” used for 61 patients in Discussion section, etc.
6. Finally, I noticed that the authors have neglected an important topic regarding the clinical course of patients with CSDHs: the “calcification/ossification of CSDH” as a rare cause of epileptic symptomatology and/or headache due to stretching of the pain-sensitive meningeal neurovascular structures, 6 7 although CSDH is frequently expected to resolve spontaneously in patients in neurosurgery.

Thus, there is a need for clarification of these points by the authors of the article.
